# Lactic acidosis switches cancer cells from aerobic glycolysis back to dominant oxidative phosphorylation

**DOI:** 10.18632/oncotarget.9746

**Published:** 2016-05-31

**Authors:** Hao Wu, Minfeng Ying, Xun Hu

**Affiliations:** ^1^ Cancer Institute (Key Laboratory For Cancer Prevention & Intervention, China National Ministry of Education), The Second Affiliated Hospital, Zhejiang University School of Medicine, Hangzhou, China

**Keywords:** Warburg effect, OXPHOS, lactic acidosis

## Abstract

While transformation of normal cells to cancer cells is accompanied with a switch from oxidative phosphorylation (OXPHOS) to aerobic glycolysis, it is interesting to ask if cancer cells can revert from Warburg effect to OXPHOS. Our previous works suggested that cancer cells reverted to OXPHOS, when they were exposed to lactic acidosis, a common factor in tumor environment. However, the conclusion cannot be drawn unless ATP output from glycolysis and OXPHOS is quantitatively determined. Here we quantitatively measured ATP generation from glycolysis and OXPHOS in 9 randomly selected cancer cell lines. Without lactic acidosis, glycolysis and OXPHOS generated 23.7% − 52.2 % and 47.8% − 76.3% of total ATP, respectively; with lactic acidosis (20 mM lactate with pH 6.7), glycolysis and OXPHOS provided 5.7% − 13.4% and 86.6% − 94.3% of total ATP. We concluded that cancer cells under lactic acidosis reverted from Warburg effect to OXPHOS phenotype.

## INTRODUCTION

Warburg effect is an enabling hallmark of cancer cell metabolism [1]. The excessive glycolysis provides cancer cells with not only ATP but also biosynthetic intermediates for rapid growth and proliferation. In contrast, normal cells have a low glycolytic rate and rely most on OXPHOS for maintaining energy homeostasis [2]. Since Warburg firstly reported the phenomenon, the switch from OXPHOS to aerobic glycolysis in cancer cells has attracted extensive attention. Its molecular basis, through yearly investigations by many researchers, has been largely unraveled. Up-regulation of glycolytic enzymes and glucose transporters via activation of Myc [3, 4], Ras [5, 6], Akt [7–9], and inactivation of p53 [10, 11] are the biochemical basis for high glycolytic rate. The switch of some glycolytic enzyme isotypes, such as switch from other PK isotypes to PKM2, also plays a part [12, 13]. Some cancer cells also exhibited Impaired mitochondria metabolism, including mutations of succinate dehydrogenase [14], fumerate hydratase [15], isocitrate dehydrogenase 2 [16–18] in Krebs cycle, and mutations in mictochondria DNA that affects respiratory chain, among others.

Despite the tremendous progress in understanding cancer cell metabolism and its regulation, the roles of small molecules in regulating cancer energy metabolism have not been extensively investigated. Lactate and proton are 2 ions commonly accumulated in tumor tissues. Lactic acidosis arises as a result of Warburg effect and the hypoxic environment further enhances glycolysis [1, 19]. The disorganized vasculature and dysfunctional capillary cause poor perfusion that permits accumulation of lactate and proton [20–23]. Hence, intratumoral lactate can reach as high as 40 mM [24] and pH as low as 6.0 [25, 26], creating a lactic acidosis condition. Lactic acidosis play multifaceted roles in tumor progression: knockdown of LDH-A diminished the tumourigenicity of cancer cells [27]; decreasing the lactate fermentation by displacing PKM2 with PKM1 reduced cancer cells' ability to form tumors in nude mice [12]; acidosis was potentially important for promoting tumour metastasis [28] and cancer progression including cancer cell metabolism [29, 30] and survival [31, 32], chromosomal instability [33, 34], and tumor angiogenesis [34, 35]. Clinical studies demonstrated that high level of lactate was a strong prognostic indicator of increased metastasis and poor overall survival [28, 29, 33, 34, 36–38].

We recently reported that lactic acidosis was a potent regulator of cancer cell glycolysis [30, 32]: in the absence of lactic acidosis, cancer cells exhibited excessive glycolysis and produced large amount of lactate; in the presence of lactic acidosis, cancer cells exhibited low glycolytic rate and produced negligible amount lactate. We also deciphered the biochemical mechanism by which lactic acidosis regulated cancer cell glycolysis [30]. Although our previous works strongly suggested that cancer cells under lactic acidosis were oxidative, this conclusion cannot be drawn, because the percentage of energy from glycolysis and OXPHOS is not known. Therefore, the purpose of this study is to quantitatively determine the percentage of ATP generation from glycolysis and OXPHOS.

## RESULTS AND DISCUSSION

**Figure 1:**
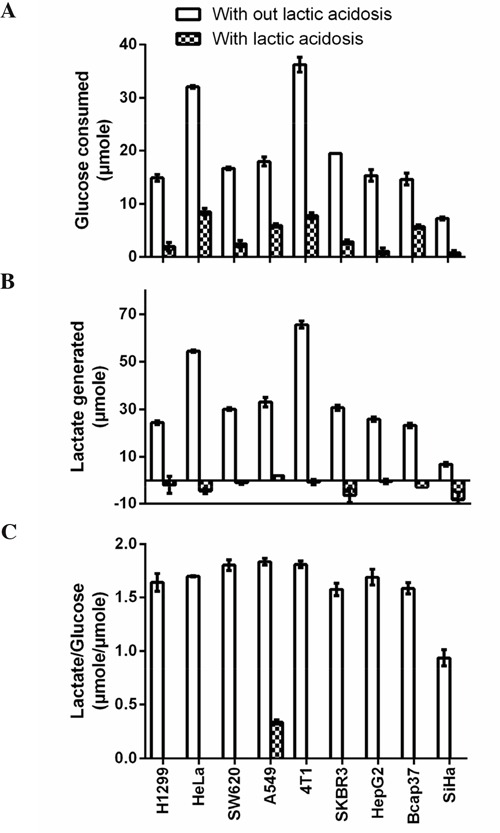
Cancer cells show typical Warburg effect Cancer cells (1 × 10^6^) were cultured in complete RPMI-1640 with or without lactic acidosis for 24 hours and glucose consumption and lactate generation were measured. **A.** Glucose consumption. **B.** Lactate generation. **C.** Lactate generated over glucose consumed. One molecule of glucose, if completely converted to lactate, will generate 2 molecules of lactate. This ratio reflects how many percentage of incoming glucose is converted to lactate. Note that under lactic acidosis, except A549, lactate generation in other cell lines were negative, meaning these cancer cells consumed lactate in medium, hence there were no ratio (lactate generated/glucose consumed) for these cells. Data are mean ± SD. Results were repeated by 3 independent experiments.

We randomly picked 9 cancer cell lines from different organ origin, so that the results could reflect general traits of cancer cells. All these cell lines, except SiHa, showed typical Warburg phenotype, as they excessively consumed glucose and converted 79 to 92% incoming glucose to lactate, as calculated according to the lactate generated/glucose consumed ratio (Figure 1). SiHa cells were relatively oxidative [29] and our data also showed that this cell line consumed smallest amount of glucose and generated least lactate among 9 cell lines (Figure 1). When these cells were cultured under lactic acidosis, glucose consumptions were dramatically reduced. Furthermore, except A549 which generated a little amount of lactate, other cells consumed lactate in culture medium, albeit to a negligible extent. These results were consistent with our previous reports [30, 32, 33, 39].

### The contribution of OXPHOS and glycolysis to ATP production without lactic acidosis

**Figure 2:**
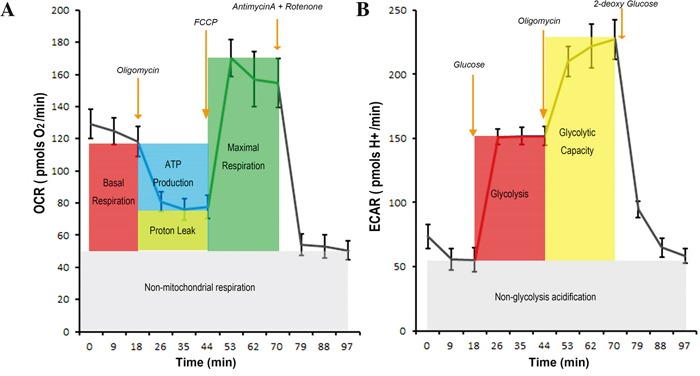
The procedure to measure the OXPHOS-linked oxygen consumption rate and glycolysis-linked proton production rate and the quality controls **A.** Measurement of OXHPHOS-linked oxygen consumption rate (OCR). H1299 Cancer cells were incubated in Seahorse-XF assay medium and the basal OCR value (summation of OXPHOS-linked OCR, the OCR associated with proton leak along respiratory chain, and the OCR irrelevant to mitochondial respiration) were obtained. When oligomycin A, an inhibitor of ATP synthase, was added, OXPHOS-associated OCR, but not the other two, was blocked. Addition of FCCP, a chemical uncoupler of electron transport and oxidative phosphorylation, maximized electron flow along the respiratory chain and the oxygen consumption. Finally, rotenone (an inhibitor of respiratory complex I) and antimycin (an inhibitor of respiratory complex III) were added to completely stop electron transfer along respiratory chain hence blocked OCR associated with ATP synthesis and proton leak. The remaining OCR was irrelevant to activity of mitochondrial respiration. **B.** Measurement of glycolysis-linked proton production rate (PPR). H1299 cancer cells were cultured in Seahorse-XF basal medium deprived of glucose and the basal proton production rate was obtained. Addition of glucose increased PPR and this increment represented glycolysis-associated PPR. Further addition of oligomycin maximized PPR because of Pasteur effect. Finally, 2-DG was added and the PPR was returned to the basal level hence glycolysis-associated PPR was confirmed. These procedures with proper quality controls were used to measure OXPHOS-associated OCR and glycolysis-associated PPR throughout this study.

In this study, we used Seahorse XF analyser to measure glycolysis - linked proton generation rate by precisely excluding non-glycolysis associated proton generation and to measure OXHPHOS-linked oxygen consumption by accurately excluding non-OXPHOS linked oxygen comsumption (Figure 2).

**Figure 3:**
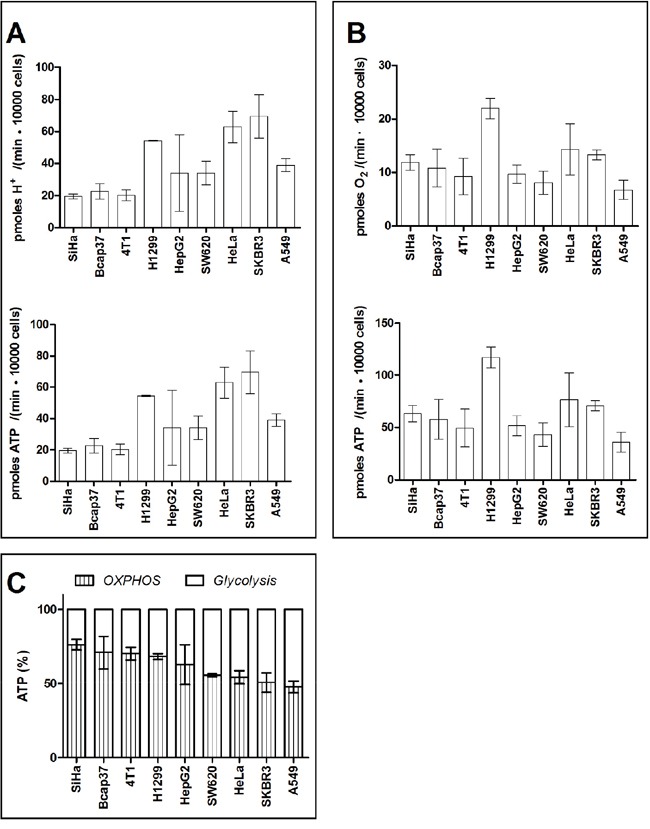
ATP generated by glycolysis and OXPHOS in cancer cells **A.** Glycolysis-associated proton generation rate and ATP generation rate. **B.** OXPHOS-linked oxygen consumption rate and ATP generation rate. **C.** Percentage of ATP generated by OXPHOS and glycolysis. Data are mean ± SD (n=16) combined from 2 independent experiments.

To calculate ATP output from glycolysis, the extracellular acidification rate (ECAR) was measured (Figure 3A upper panel). The ECAR was then converted to proton production rate (PPR). At steady-state glycolysis, the stoichiometry between PPR and ATP output rate is 1. Therefore, ATP production rates from glycolysis in 9 cancer cell lines were between 19.5-69.4 pmoles / (min ·10000 cells) (Figure 3A low panel).

To calculate ATP output from OXPHOS, OCR of the 9 cancer cell lines were measured (Figure 3B upper panel). At steady-state oxidative phosphorylation, the stoichiometry between OCR and ATP output rate is 5-5.3 (complete oxidation of 1 mole glucose generates 30-32 mole ATP). OCR by these 9 cancer cell lines were between 6.8 − 22.1 pmoles O_2_ / (min ·10000 cells). Therefore, ATP production rate from OCR in 9 cancer cell lines were between 36.0-117.1 pmoles / (min ·10000 cells) (Figure 3B low panel).

Taken together, when cells exhibited Warburg phenotype, glycolysis generated 23.7-52.2% ATP, whereas OXPHOS provided 47.8-76.3% ATP (Figure 3C).

The data are agreeable with the Warburg's measurement. Warburg indicated in his ‘On the Origin of Cancer cells’ [40] that “This, converted to energy equivalents, means that the cancer cells can obtain approximately the same amount of energy from fermentation as from respiration, whereas the normal body cells obtain much more energy from respiration than from fermentation”. In this study, OXPHOS and glycolysis provided comparable rates of ATP in SW620, Hela, SKBR3, and A549.

In the remaining 5 cell lines, OXPHOS provided more ATP than glycolysis. This is not surprising, as it was reported that OXPHOS provided more ATP than glycolysis in HT29 [41] and MCF-7 [42], 2 cancer cell lines with Warburg phenotype.

Taken together, the data agrees with the previous reports.

### Contribution of OXPHOS and glycolysis to ATP production in the presence of lactic acidosis

**Figure 4:**
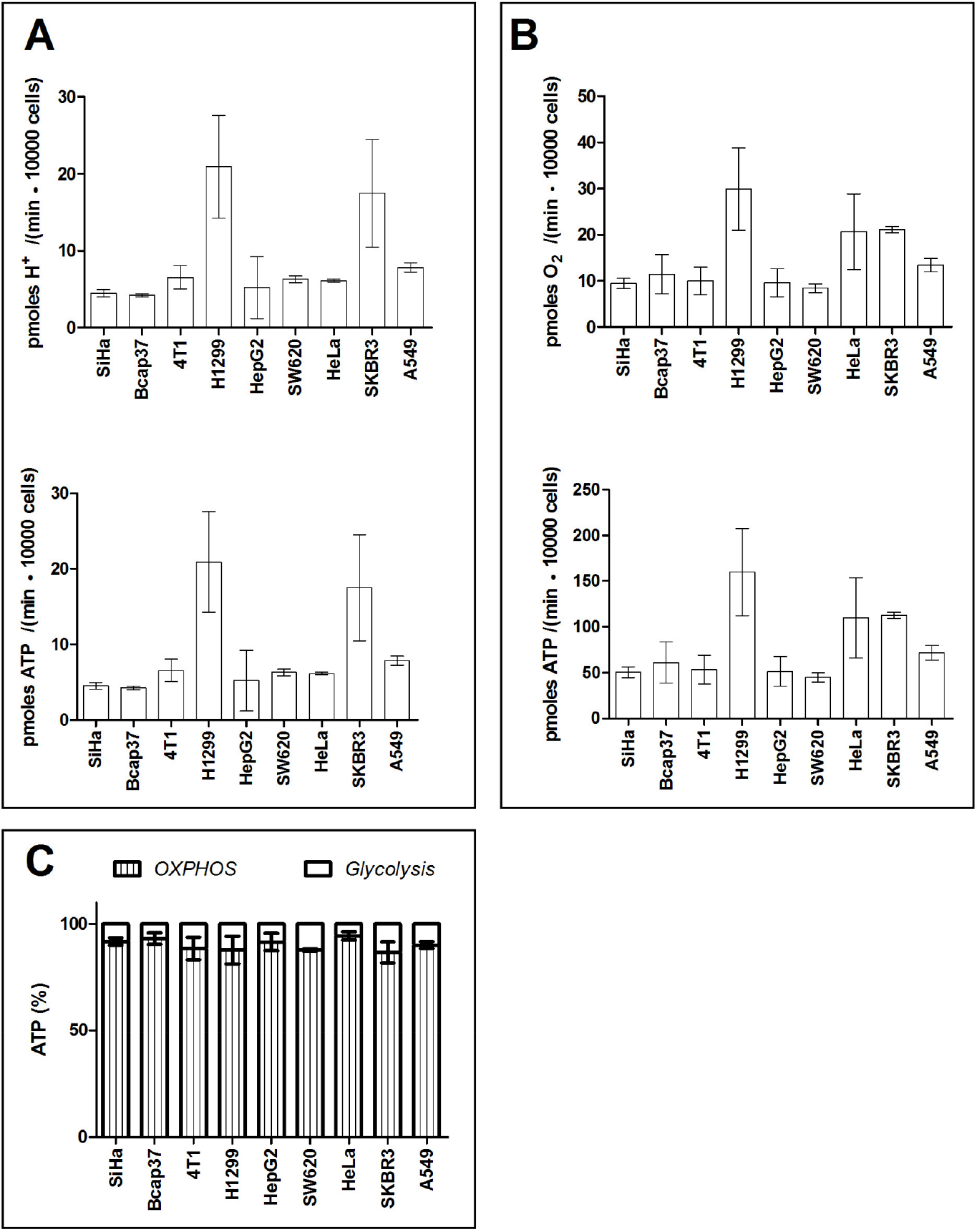
Percentage of ATP contributed by glycolysis and OXPHOS in cancer cells in Seahorse-XF medium supplemented with lactic acidosis **A.** Glycolysis-associated proton generation rate and ATP generation rate. **B.** OXPHOS associated-oxygen consumption rate and ATP generation rate. **C.** Percentage of ATP generated by OXPHOS and glycolysis. Data are mean ± SD (n=16). Results were repeated by 2 independent experiments.

Then we measured the percentage of ATP output by glycolysis and OHPHOS in cancer cells under lactic acidosis. The lactic acidosis condition (20 mM lactate and pH 6.7) is an optimal condition refined by us before [30, 32, 33, 43]. Under such condition, cancer cells markedly reduced glycolysis rate and generated no or negligible amount of lactate. The proton production rate by 9 cancer cell lines were 4.2-20.9 pmoles H^+^ / (min ·10000 cells), equivalent to ATP output rate of 4.2-20.9 pmoles ATP / (min ·10000 cells) (Figure 4A). OCR by these 9 cancer cell lines were 8.5-30.0 pmoles O_2_ / (min ·10000 cells) cells, equivalent to ATP output rate of 45.1-159.0 pmoles ATP / (min ·10000 cells) (Figure 4B). Thus, when cancer cells were under regular culture with lactic acidosis, glycolysis and OXPHOS in these cell lines accounted for 5.7 − 13.4% and 86.6 − 94.3 % of total generated ATP, respectively (Figure 4C). Notably, 9 tested cancer cell lines without an exception exhibited oxidative phenotype.

The underlying mechanism for lactic acidosis to revert cancer cells from aerobic glycolysis to OXPHOS was deciphered in our previous publication [30, 39]. First, under regular culture, the cytosolic pH was 7.3 and the cytosolic lactate was around 2 mM, whereas under lactic acidosis (20 mM lactate, pH6.6), the cytosolic pH decreased to 6.9, and cytosolic lactate increased to 20 mM [30]. Second, the 0.4 unit decrease of pH dramatically reduced the glycolysis rate, because glycolytic enzymes including HK and PFK1 and glucose transporter were sensitive to proton inhibition [30]. Third, the increase of cytosolic lactate brought the mass action ratio of lactate dehydrogenase(LDH)-catalyzed reaction approaching to equilibrium constant, thereby the forward and backward rate of the reaction was nearly equal [30, 39] (note that cytosolic pyruvate concentration and the cytosolic free [NAD]/[NADH] kept nearly constant hence the lactate concentration became the major variable of mass action ratio of this reaction).

Two additional points should be mentioned. First, when culture condition changed from lactic acidosis condition to regular culture, cancer cells instantly switch from non-Warburg phenotype to Warburg phenotype [30]. Second, lactic acidosis did not suppress the expression of glycolytic enzymes and glucose transporter [30].

Taken together, the switch between Warburg and non-Warburg phenotype under lactic acidosis is simply due to the proton effect on glycolysis and lactate concentration on the LDH-catalyzed reaction status (equilibrium or non-equilibrium).

As aerobic glycolysis was considered important for survival and proliferation of cancer cells, would lactic acidosis affect cancer cell growth? First, lactic acidosis is an intratumoral factor and it is not toxic to cancer cells. Second, cancer cells under lactic acidosis can progressively grow [30, 32, 33, 43]. Third, lactic acidosis is particularly important for cancer cells when glucose supply is a problem [30, 32]: when 4T1 cells were cultured with 0.5 mM glucose, the number of 4T1 cells were tripled in 3 days in the presence of lactic acidosis, but died out in the absence of lactic acidosis. Moreover, when glucose was deprived, cancer cells (4T1, Bcap37, RKO, SGC7901) died out within 3 days, but could survive between 30 to 70 days in the presence of lactic acidosis. Taken together, lactic acidosis confers cancer cells with an economic way to use glucose and with resistance to glucose deprivation-induced death. The current study provides quantitative data to support our notion, that cancer cells can switch back and forth between typical Warburg and oxidative phenotype.

Cancer cells primarily depending on OXPHOS under lactic acidosis may have translational implication. Many previous studies suggested that targeting mitochondria could be an alternative way to treat cancer [44, 45]. Given that the lactic acidosis is common in many solid tumors, and given that cancer cells under lactic acidosis primarily rely on OXPHOS for ATP generation, targeting mitochondria metabolism may have potential to control tumor.

## MATERIALS AND METHODS

### Cells

Nine randomly chosen cell lines, 4T1, Bcap37, HepG2, HeLa, A549, H1299, SKBR3, SW620, and SiHa were maintained in RPMI-1640 medium (Invitrogen) supplemented with 10% fetal bovine serum, 100 U/ml of penicillin/streptomycin under 37°C and 5% CO_2_ incubation condition.

### Reagents

Sodium pyruvate, lactate, lactic acid, NADH, NAD^+^, ATP, hydrazine, hexokinase, G6PDH, LDH were obtained from Sigma-Aldrich (St. Louis, MO). XF Cell Mito Stress Test kit containing oligomycin, carbonyl cyanide p-trifluoromethoxyphenylhydrazone (FCCP), antimycin A and rotenone, and XF Glycolysis Stress Test kit containing oligomycin, 2-deoxyglucose (2-DG) and glucose, and unbuffered medium were purchased from Seahorse Biosciences (North Billerica, MA).

### Measurements of glucose consumption and lactate generation

1×10^6^ cells were seeded into 25cm^2^-culture flask (Corning) with complete RPMI-1640 medium to allow attachment overnight in a humidified CO_2_ incubator. The culture medium were replaced with 10 ml fresh RPMI-1640 medium with 6 mM glucose on the following day. 24 hours later, culture medium was collected for glucose and lactate concentration determination. The glucose consumption and lactate generation were determined enzymatically as described previously [32]. In the lactic acidosis condition, the culture medium was the same as above except that the medium contained 20 mM lactate with a pH 6.6. In our previous studies, we defined that under such lactic acidosis condition, cancer cells consumed glucose slowly and generated negligibly amount of lactate [30, 32, 33, 39]. Therefore, lactic acidosis condition (20 mM lactate with pH 6.6) were used throughout the study.

### Measurements of oxygen consumption rate (OCR) and proton production rate (PPR)

A Seahorse Biosciences XF96 Extracellular Flux Analyzer was used to measure the rate change of dissolved oxygen and pH in unbuffered DMEM medium immediately surrounding adherent cells cultured in a XF96 cell culture microplate (Seahorse biosciences).

Unbuffered DMEM medium with lactic acidosis (20mM lactate, pH 6.6) or without lactic acidosis (0 mM lactate, pH 7.4) were prepared, and the buffer capacity was determined by adding 0.1 N NaOH solution into the medium and monitoring the pH change. The buffering capacity is calculated as:

Buffer capacity (BC, M) = (Added NaOH volume) x (NaOH concentration) ÷ (pH change) ÷ (tested medium volume), meaning the equivalent of [OH^−^] needed to change pH by 1.0 unit in 1.0 liter of the medium.

The experiments were performed according to Seahorse XF96 manual. 1 × 10^4^ (4T1 cells) or 2 × 10^4^ (other cell lines) in 80 μL complete RPMI-1640 medium were seeded in 96 well XF Microplate (Seahorse Biosciences), and incubated at 37°C with 5% CO_2_ overnight to allowed full attachment. Then, cells were washed twice with Seahorse XF basal medium prewarmed to 37°C followed by addition of 175 μl Seahorse XF basal medium, and equilibrated at 37°C without CO_2_ for 60 minutes. The Seahorse XF analyzer used one cartridge plate with 96 optical oxygen and pH sensors to measure dissolved oxygen and pH change in 96-well microculture plate. The plungers in the cartridge plate mixed assay medium in each well for 4 minutes to allow dissolved oxygen and pH to reach equilibrium before each rate measurement. For measurement of rate, the plungers covered the cell layer, and isolated a small chamber with volume less than 3 μl. The optical sensor at the end of plunger monitored O_2_ concentration and pH periodically over 3 minutes, and the OCR and extracellular acidification rate (ECAR) value were calculated by the slope of these parameters vs. time. With the predetermined buffer capacity, Seahorse XF analyzer calculated proton production rate (PPR). After measurement, the plungers ascended, and the media were re-mixed to allow re-equilibrium. Baseline rate were measured 3 times. According to the manufacturer's instruction, the cell density gives an OCR between 50-150 pMoles/min and consistent monolayer was considered optimal. We adjusted the number of cells to fit the window of OXPHOS rate. We did cell titration using 4T1 cells (Supplementary Figure 1) For other cells, we did not do cell titration, but simply fit the OXPHOS rate into the measurement window recommended by the manufacturer.

For OCR measurement, the assay medium used was unbuffered DMEM medium (Seahorse biosciences), supplemented with 10mM Glucose and 1mM pyruvate, with or without lactic acidosis. The oligomycin, FCCP, antimycin/rotenone were preloaded into the delivery chambers in the cartridge plate and sequentially injected into each well at the optimized concentration. The last baseline value and the most changed value after each injection were taken for data analysis. The difference between baseline OCR and oligomycin response OCR is the oxygen consumption related to ATP production: OCR_basal_ − OCR_oligomycin_=OCR _ATP production_

For glycolysis rate determination, the assay medium used was XF base medium (Seahorse Biosciences), supplemented with 2 mM glutamine, with or without lactic acidosis. Glucose, oligomycin and 2-DG were injected into each well sequentially. The last PPR value before chemical injection was taken as the basal PPR (PPR_basal_), and the biggest PPR after glucose addition was taken as the response to glucose (PPR_glucose_). The glycolysis related PPR (PPR_glycolysis_) = PPR_glucose_ − PPR_basal_.

In the procedure of OCR measurement, when the oxidative phosphorylation uncoupler FCCP is added, oxygen consumption by the respiratory chain was resumed, when rotenone (complex I inhibitor) and antimycin A (complex III inhibitor) were added, the electron transfer as well as oxygen consumption by the respiratory chain was blocked. This confirmed that oligomycin-blocked oxygen consumption was associated with OXPHOS. In the procedure of ECAR measurement, oligomycin addition enhanced ECAR, a reversed Pasteur Effect, and 2-DG addition blocked proton production, confirming that proton generation was associated with glycolysis.

After determination, 3 parallel wells of cells were counted to normalize the measured data.

### Statistics

The statistical difference was tested using unpaired 2-tailed t-test.

## SUPPLEMENTARY DATA FIGURE


