# Supplementary material for: Lactic acidosis switches cancer cells from aerobic glycolysis back to dominant oxidative phosphorylation
Source: Oncotarget. 2016 May 31;7(26):40621–9. doi: 10.18632/oncotarget.9746 (PMC5130031; doi:10.18632/oncotarget.9746)
Supplement: Supplementary file 1 [file oncotarget-07-40621-s001.pdf]

Lactic acidosis switches cancer cells from aerobic glycolysis back to dominant oxidative phosphorylation

SUPPLEMENTARY DATA

Detailed protocol for ECAR and OCR measurement

A: OCR measurement:

1. Day1:

a) Seeding desired number of cells in to the 96 well XF96 cell culture Microplate in 80 µL complete medium, and incubate the plate at 37°C with 5% CO2 overnight;
b) Add 200 µL XF96 Calibrant (pH 7.4) to each well of a 96-well Utility plate; and then place XF Sensor Cartridge into each well of the plate. Make sure that all the probes on the Senor Cartridge are immersed in the calibrant. There is a single sensor spot with fluorescent chemicals on the top of probe to detect OCR and ECAR;
c) Add 10mM glucose and 1mM pyruvate into the Seahorse XF Assay medium to get the assay medium for OCR.

2. Day2:

a) Warm the assay medium to 37°C;
b) Change the medium in the cell culture plate to 175µL warmed assay medium, change the medium 3 time;
c) Incubate the cell culture plate in a 37°C incubator without CO2 for 1 hour to allow cells to equilibrate to the new medium;
d) Add 25µL 8X oligomycin, 9X FCCP and 10X antimycin/rotenone in the Injection Port A, B, and C of each probe on the XF Cartridge, respectively;
e) After setting the experimental template on the XF96 software, load the hydrated sensor cartridge with the calibrant plate into the XF96 Analyzer. It will take about 30 minutes to finish the calibration;
f) After calibration is complete, the Analyzer prompts out the calibration plate. Remove the plate and place the cell culture plate;
g) The Analyzer will inject the oligomycin in all A Ports at once by compressed air at the planed time point, as well as all B and C ports;
h) The OCR is measured 3 times before and after each injection.
i) The measurements before oligomycin injection are the basal OCR.

B: ECAR measurement:

1. Day1:

a) Seeding desired number of cells in to the 96 well XF96 cell culture Microplate in 80 µL complete medium, and incubate the plate at 37°C with 5% CO2 overnight;
b) Add 200 µL XF96 Calibrant (pH 7.4) to each well of a 96-well Utility plate; and then place XF Sensor Cartridge into each well of the plate. Make sure that all the probes on the Senor Cartridge are immersed in the calibrant. There is a single sensor spot with fluorescent chemicals on the top of probe to detect OCR and ECAR;
c) Add 2mM glutamine into the Seahorse XF base medium to get the assay medium for ECAR.

2. Day2:

a) Warm the assay medium to 37°C;
b) Change the medium in the cell culture plate to 175µL warmed assay medium, change the medium 3 time;
c) Incubate the cell culture plate in a 37°C incubator without CO2 for 1 hour to allow cells to equilibrate to the new medium;
d) Add 25µL 8X glucose, 9X oligomycin and 10X 2-Deoxy-glucose in the Injection Port A, B, and C of each probe on the XF Cartridge, respectively;
e) After setting the experimental template on the XF96 software, load the hydrated sensor cartridge with the calibrant plate into the XF96 Analyzer. It will take about 30 minutes to finish the calibration;
f) After calibration is complete, the Analyzer prompts out the calibration plate. Remove the plate and place the cell culture plate;
g) The Analyzer will inject the oligomycin in all A Ports at once by compressed air at the planed time point, as well as all B and C ports;
h) The ECAR is measured 3 times before and after each injection.
i) The measurements before oligomycin injection are the basal ECAR.

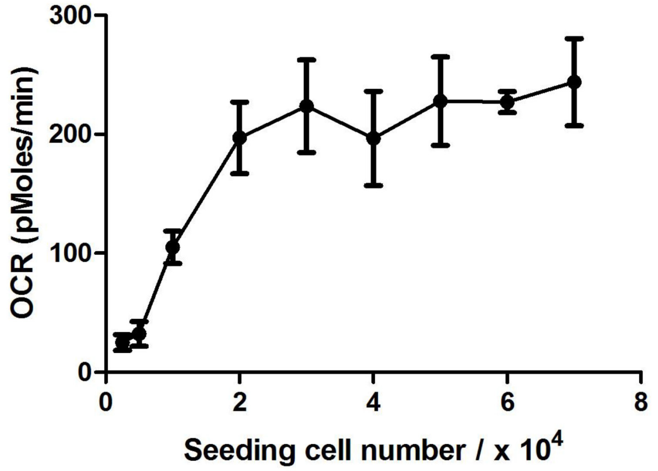

Supplementary Figure S1: Cell number titration of 4T1 cells. 4T1 cell was seeded in different numbers, and the basal OCR was measured.
